# Pathophysiology of Major Depression by Clinical Stages

**DOI:** 10.3389/fpsyg.2021.641779

**Published:** 2021-08-05

**Authors:** Ana Cecília de Menezes Galvão, Raíssa Nobrega Almeida, Geovan Menezes de Sousa, Mario André Leocadio-Miguel, Fernanda Palhano-Fontes, Dráulio Barros de Araujo, Bruno Lobão-Soares, João Paulo Maia-de-Oliveira, Emerson Arcoverde Nunes, Jaime Eduardo Cecilio Hallak, Felipe Barreto Schuch, Jerome Sarris, Nicole Leite Galvão-Coelho

**Affiliations:** ^1^Postgraduate Program in Psychobiology, Laboratory of Hormone Measurement, Department of Physiology and Behavior, Federal University of Rio Grande do Norte, Natal, Brazil; ^2^Laboratory of Neurobiology and Biological Rhythms, Department of Physiology and Behavior, Federal University of Rio Grande do Norte, Natal, Brazil; ^3^Brain Institute, Federal University of Rio Grande do Norte, Natal, Brazil; ^4^National Institute of Science and Technology in Translational Medicine, São Paulo, Brazil; ^5^Department of Biophysics and Pharmacology, Federal University of Rio Grande do Norte, Natal, Brazil; ^6^Department of Clinical Medicine, Federal University of Rio Grande do Norte, Natal, Brazil; ^7^Department of Psychiatry, Federal University of Rio Grande do Norte, Natal, Brazil; ^8^Department of Neurosciences and Behavior, University of São Paulo, São Paulo, Brazil; ^9^Department of Sports Methods and Techniques, Federal University of Santa Maria, Santa Maria, Brazil; ^10^NICM Health Research Institute, Western Sydney University, Westmead, NSW, Australia; ^11^Professorial Unit, The Melbourne Clinic, Department of Psychiatry, University of Melbourne, Parkville, VIC, Australia

**Keywords:** cortisol, C-reactive protein, brain-derived neurotrophic factor, sleep quality, precision psychiatry

## Abstract

The comprehension of the pathophysiology of the major depressive disorder (MDD) is essential to the strengthening of precision psychiatry. In order to determine the relationship between the pathophysiology of the MDD and its clinical progression, analyzed by severity of the depressive symptoms and sleep quality, we conducted a study assessing different peripheral molecular biomarkers, including the levels of plasma C-reactive protein (CRP), serum mature brain-derived neurotrophic factor (mBDNF), serum cortisol (SC), and salivary cortisol awakening response (CAR), of patients with MDD (*n* = 58) and a control group of healthy volunteers (*n* = 62). Patients with the first episode of MDD (*n* = 30) had significantly higher levels of CAR and SC than controls (*n* = 32) and similar levels of mBDNF of controls. Patients with treatment-resistant depression (TRD, *n* = 28) presented significantly lower levels of SC and CAR, and higher levels of mBDNF and CRP than controls (*n* = 30). An increased severity of depressive symptoms and worse sleep quality were correlated with levels low of SC and CAR, and with high levels of mBDNF. These results point out a strong relationship between the stages clinical of MDD and changes in a range of relevant biological markers. This can assist in the development of precision psychiatry and future research on the biological tests for depression.

## Introduction

The burden of mental disorders has been increasing worldwide (Vigo et al., 2016; World Health Organization(Who), 2017). Major depressive disorder (MDD) is one of the most prevalent mood syndrome, affecting 350 million people globally (World Health Organization(Who), 2017; Liu et al., 2020). Currently, there is a thought in the research field that the comprehension of MDD pathophysiology is essential to the strengthening of precision psychiatry, by supporting prognosis, diagnosis, treatments, and follow-up of patients, and thus would help in the reduction of MDD burden (Schmidt et al., 2011; Dean and Keshavan, 2017; Menke, 2018). These are the main goals of the large European and American studies such as the Roadmap for Mental Health Research and Research Domain Criteria, respectively (Insel et al., 2010; Elfeddali et al., 2014).

Many studies have tried to associate neurobiological markers with a set of MDD symptoms as shown in *5th Diagnostic and Statistical Manual of Mental Disorders* (DSM-5) (American Psychiatric Association, 2013; Hashimoto, 2015; Fried et al., 2019; Koo et al., 2019). However, the physiological dysfunctions observed in patients with major depression are inconsistent: they are not present in all patients with the same intensity, and with similar relationship with symptoms (Bremmer et al., 2017; Zou et al., 2018; Almeida et al., 2019). Therefore, at present, none biomarker validated in scientific studies is widely used clinically (Menke, 2018). It is suggested that the progression of clinical disease, such as the severity of depressive symptoms and the sleep changes, and some sociodemographic characteristics, like gender, age, income, and education, can be related to the large variance observed in the pathophysiology of patients with depression (Mello et al., 2007; Verduijn et al., 2015; Menke, 2018; Kim, 2020; Perna et al., 2020; Santiago et al., 2020).

Some patients with MDD often show dysregulation of hypothalamus-pituitary-adrenal (HPA) axis, which is the main endocrine stress system. However, the studies have conflicting views about up- and downregulation of HPA axis in these patients (Bremmer et al., 2017; Dean and Keshavan, 2017; Ferrari and Villa, 2017). Some show systemic hypercortisolemia (Gold et al., 1986, 1988), while other studies show hypocortisolemia (Tu et al., 2013; Kunugi et al., 2015). Furthermore, disruptions in the cortisol awakening response (CAR) and the circadian cortisol pattern are also found, which can be measured from saliva samples (Chida and Steptoe, 2009; Marques et al., 2010; Vreeburg et al., 2013; Moreira et al., 2016; Galvão et al., 2018).

Increased systemic inflammation is another physiological alteration observed in some patients with depression (Krishnadas and Cavanagh, 2012; Lopresti et al., 2014; Zou et al., 2018). It is proposed that cortisol changes disrupt homeostasis of the immune system, stimulating proinflammatory cytokines, such as interleukin 6 (IL-6), and tumor necrosis factor-α (Zunszain et al., 2011; Sousa et al., 2015). Furthermore, an increase in the C-reactive protein (CRP) levels, a non-specific inflammatory biomarker released by hepatocytes, is also observed in patients with MDD (Haapakoski et al., 2015; Köhler-Forsberg et al., 2016; Galvão-Coelho et al., 2020).

In addition, neuroimaging studies have revealed a reduced hippocampal volume in some patients with depression, which is speculated to be related to a decrease in the levels of neurotrophins, such as brain-derived neurotrophic factor (BDNF) (Lima-Ojeda et al., 2018; Roddy et al., 2019; Sheline et al., 2019). Although some studies have shown lower levels of blood BDNF in patients with major depression (MD) in comparison with those of healthy subjects, the results are inconsistent (Karege et al., 2002; Groves, 2007; Lee and Kim, 2009; Vu and Student, 2009; Cubala and Landowski, 2014; Almeida et al., 2019).

Interestingly, all these changes, including HPA axis, immune system, and the levels of BDNF, have physiological pathways that are often integrated with sleep disturbances in a disrupted positive-feedback system (Cubala and Landowski, 2014; Giese et al., 2014; Kunugi et al., 2015; Chrousos et al., 2016; Ferrari and Villa, 2017; van Dalfsen and Markus, 2018). The decrease in sleep quality is usually observed before the onset of MDD (Breslau et al., 1996; Perlis et al., 1997; Ohayon and Roth, 2003), while persistent sleep impairments are associated with the severity of MDD, worse treatment outcomes, and consequently, the recurrence of illness (Moos and Cronkite, 1999; Santiago et al., 2020).

Despite the knowledge about the relationship across these important biological systems, a minor number of studies have evaluated these changes simultaneously and over the clinical progression of MDD (Verduijn et al., 2015). Therefore, the purpose of this study was to evaluate patients with depression in distinct MDD stages (*de novo* patients and patients with treatment-resistant depression), by the serum mature BDNF (mBDNF), plasma CRP, serum cortisol (SC), and the salivary CAR, aiming to compare them with those of a control group of healthy volunteers. These physiological parameters were analyzed by taking into account the severity of the depression symptom, sleep quality, and sociodemographic characteristics of the participants. We hypothesized that patients with depression have distinct physiological profiles from healthy volunteers, which will be influenced by the stage of disease, that is, worse physiological parameters would be related to stronger depressive symptoms and worse sleep quality.

## Materials and Methods

### Ethical Aspects

This study was approved by the Medical Research Ethics Committee of the Onofre Lopes University Hospital (HUOL) (# 579,479) and by the Human Research Ethics Committee of the Federal University of Rio Grande do Norte (UFRN) (# 2.628.202). This study was registered at http://clinicaltrials.gov (NCT02914769/U1111-1215-4472). The procedures of this study comply with the ethical standards of the relevant national and institutional committees for human experimentation and with the Declaration of Helsinki of 1975, revised in 2008. The research was carried out at UFRN. Individuals who met inclusion and exclusion criteria were invited to voluntarily participate in this study. They signed the informed consent that provided information about the study and granted complete freedom to drop out from the study at any time. All information collected in this study was kept confidential.

### Participants

The recruitment of adult participants of both sexes was performed by psychiatrist referrals at local outpatient psychiatric units and by advertising on local and social media between 2016 and 2018. The sample size was determined for G*Power (version 3.1.9.4) (Faul et al., 2009), so 120 volunteers were required to reach an effect size of 1.13. All volunteers had a full clinical evaluation by a trained psychiatrist which included anamnesis, mental health, and neurological health evaluation using the Structured Clinical Interview for Axis I (SCID; DSM-IV) and Hamilton Depression Scale (HAM-D) (Hamilton, 1960). After the screening, volunteers were allocated into the following groups:

*Patient group* (*n* = 58): Volunteers diagnosed with MDD on the SCID who were in the course of a depressive episode. The severity of the disorder was assessed by HAM-D. Patients with a current or earlier diagnosis of drug abuse or substance-related disorder, schizophrenia, bipolar affective disorder, mania or hypomania, or neurological and inflammatory disorders, such as autoimmune or infectious disease, were not included in this study. In addition, the diagnosis of personality disorders was assessed by a clinical interview with a psychiatrist guided by the criteria instrument of the *4th Diagnostic and Statistical Manual of Mental Disorders* (DSM IV-TR). Patients were grouped according to an initial depression diagnosis (*de novo* patients) or treatment-resistant depression (TRD), as follows:

• Patients with the first episode of MD (*n* = 30; 14 men and 16 women): Patients who got their first diagnosis of MD in the clinical evaluation of this study and who were naïve to antidepressants drugs. During this study, these patients were free of medications with effects on cognition and mood, and on neurovegetative, immune, and endocrine functions. According to the HAM-D, the severity of MDD in these patients ranged from mild to moderate.
• Patients with TRD (*n* = 28; 7 men and 21 women): Patients who did not respond to at least two earlier classes of antidepressants (Hamilton, 1960). During this study, these patients were under a 15-day washout period, i.e., without any antidepressant use, since they were in the process of changing their antidepressant medication. The use of benzodiazepines, hypnotic drugs, and/or anxiolytic drugs under prescription was allowed. According to the HAM-D, the severity of MDD in these patients ranged from moderate to severe.

*General control group* (*n* = 62): Healthy volunteers without the diagnosis of physical, sleep, neurological, or psychiatric disorders and who were free of medications with effects on cognition and mood, and on neurovegetative, immune, and endocrine functions. They were grouped into the following subgroups:

• Control group 1 (CG1: *n* = 32; 15 men and 17 women): Volunteers paired with sociodemographic characteristics of patients with a *de novo* diagnosis of MD group.
• Control group 2 (CG2: *n* = 30; 7 men and 23 women): Subjects paired with sociodemographic characteristics of the patients with treatment-resistant depression.

### Experimental Design

The night before the data collection, volunteers slept in the laboratories of sleep at UFRN, when their height and body weight were measured to calculate the body mass index (BMI), and the Pittsburgh Sleep Quality Index (PSQI) was assessed. On the following day, around 6:00 a.m., saliva samples were collected, followed by blood collection. All volunteers were fasting for approximately 8 h.

### Psychometric Scales

Hamilton Depression Scale (Conway et al., 2017) consists of a semistructured interview for the identification of frequency and intensity of depressive symptoms assessed by a trained psychiatrist (Rush et al., 2004; Howland, 2008). HAM-D has 17 sub-domains, namely, depressed mood (H1), feelings of guilt (H2), suicide (H3), initial insomnia (H4), insomnia during the night (H5), delayed insomnia (H6), work and interests (H7), retardation (H8), agitation (H9), psychiatric anxiety (H10), somatic anxiety (H11), gastrointestinal somatic symptoms (H12), general somatic symptoms (H13), genital symptoms (H14), hypochondriasis (H15), weight loss (H16), and insight (H17). According to the HAM-D scores, patients are clustered into one of the three following categories: (1) mild: 10 ≤ score ≤ 13; (2) moderate: 14 ≤ score ≤ 17; and (3) severe: scores > 17 (Halfaker et al., 2011).

Pittsburgh Sleep Quality Index is a self-reported instrument used to assess sleep quality and disturbances over a 1-month time interval (Buysse et al., 1989; Bertolazi et al., 2011). It has seven sub-domains, namely, subjective sleep quality (C1), sleep latency (C2), sleep duration (C3), habitual sleep efficiency (C4), sleep disorder (C5), sleep medication use (C6), and daytime sleep dysfunction (C7). This tool has an overall score ranging from 0 to 21 points, which can be categorized into good sleep (0–4 points), poor sleep (5–10 points), and sleep disorder (greater than 10 points).

### Saliva and Blood Collections

Three saliva collections were performed by a trained researcher, using a Salivette (Sarstedt), a plastic tube, and a cotton piece specifically used for saliva collection. The first collection was performed at awakening, then 30 min later, and the third, 45 min later. Before or during saliva collection, volunteers were instructed not to rinse their mouths, not to eat or drink, and to remain lying in the bed. Later, blood collection was performed by a nurse or a trained researcher using perforating and disposable material (needle and syringe).

### Biochemical Dosage

Saliva was used to measure cortisol by using ELISA DRG-SLV 4635 kit. Blood was used to measure serum cortisol by using ELISA kit DRG 1887 and mBDNF by using ELISA kit BDNF SK00752-01 (Aviscera Bioscience). Moreover, CRP was assessed by using two distinct tests. The first one was the agglutination plate of latex CRP of EBRAM, which suggests the presence or absence of inflammation. In the second test, the levels of serum CRP were assessed by using immunoturbidimetry. All measurements were performed blindly, for groups and individuals, and in duplicate. In this study, the intra- and inter-assay coefficients of variation were 3.97 and 13.01% for SC, 4.78 and 16.30% for salivary cortisol, 6.15 and 21% for mBDNF, and 2 and 3.3% for CRP, respectively.

### Statistical Analysis

The physiological parameters (e.g., CAR, SC, mBDNF, and CRP), PSQI, and HAM-D (both total score and the sub-score of PSQI and HAM-D sub-domains selected by random forest–based algorithm, see below) were considered quantitative-dependent variables, while the groups (i.e., MD, TRD, CG1, and CG2) were considered the categorical-independent variables. The sociodemographic characteristics, such as gender (i.e., categorical variable), age (i.e., continuous quantitative variable), BMI (i.e., metric quantitative variable), income (i.e., categorical variable), and education (i.e., categorical variable), were used as covariates.

For CAR, its area under the curve was calculated using the three saliva sample points (Pruessner et al., 2003). CAR, SC, HAM-D, and sub-score of HAM-D (sHAM-D) were log-transformed to reach Gaussian distribution. Outliers were considered as values with 3 SDs below or above the mean (for Gaussian variables) or outside the quartile 25–quartile 75 range (for non-Gaussian variables) and were excluded from the analysis.

The statistical plan comprised four sets of analyses; the significance level considered was *p* ≤ 0.05 for all tests. First, we applied the Boruta test, which is a random forest–based algorithm that selects variables based on its importance score in comparison with a shuffled attribute (Kursa and Rudnicki, 2010). Here, we used it to rank the relevance of the sociodemographic characteristics of volunteers for the discrimination of groups MD × CG1 and TRD × CG2 separately (relevant characteristic must score above a shuffled data, *I* > 5.94). This algorithm was also applied to HAM-D and PSQI components, aiming to select those most relevant to discriminate between patient groups (MD and TRD). Those selected components were grouped for constructing sHAM-D (*I* > 4.24) and sub-score of PSQI (sPSQI; *I* > 4.93), respectively. The default hyperparameters were used in this test (Buysse et al., 1989). Numerical variables were previously standardized in *z*-score.

Following, in the second set of analyses, we used some multivariate tests aiming to find differences in physiological parameters between depressive groups and its control. We applied the MANCOVA test to physiological parameters with the Gaussian distribution (i.e., CAR, SC, and mBDNF), whereas the General Linear Model (GLM) was used to analyze the non-Gaussian variables (i.e., CRP) between groups. In each one of these tests, the analyses were done separately for MD × CG1 and for TRD × CG2. The *p*-value correction for multiple tests and bootstrap was applied. In both analyses (i.e., MANCOVA and GLM), the relevant sociodemographic characteristics previously selected by using the Boruta test were included as covariates. When a covariate was significant in multivariate analysis, we separately applied the Spearman’s correlation test (for quantitative covariates) or the independent *t*-test (for qualitative covariates), aiming to find the relationship between the covariate and the respective dependent variable. Cohen’s *d* effect size and its 95% CI for group comparisons are reported.

The third set of analyses corresponded to the correlation analyses, which were performed by merging the MD and TRD groups as a single group of patients with depression. The Spearman’s correlation test was performed to analyze the numerical physiological parameters in relation to the severity of the disease (measured by HAM-D and sHAM-D) and sleep quality (measured by PSQI and sPSQI). Although age was significantly different between the groups of depressive patients (Mann–Whitney *U* = 88, *p* < 0.001, TRD μ = 41.57 ± 11.61, and MD μ = 24.2 ± 3.84), the sociodemographic characteristics were not controlled as covariables since this test does not support it.

The fourth set of analyses is the predictive models. We performed linear regressions to investigate whether the earlier use of antidepressant treatment, as a dummy variable, could predict the physiological parameters (i.e., CAR, SC, and mBDNF).

## Results

From the initial inquiries of 640 participants, 120 participants who potentially met the inclusion criteria were selected for the screening phase: 62 healthy participants and 58 patients with MD, with 30 being grouped as patients with first episode of MD (14 men and 16 women) and 28 participants with TRD (7 men and 21 women). The healthy participants were divided into two groups, namely, a control group (CG1, *n* = 32; 15 men and 17 women) for the *de novo* MD group and a control group for TRD (CG2, *n* = 30; 7 men and 23 women). The consolidated standards for clinical trial reports (CONSORT) are shown in Supplementary Figure 1.

### Pathophysiology of Patients With Depression Versus Healthy Volunteers

#### Patients With First Episode Depression (*de novo* Patients)

All patients (first depressive episode, MD: *n* = 30) and their respective control group (CG1: *n* = 32) were Brazilian and young adults (MD: 24.2 ± 3.84; CG1 = 27.06 ± 6.42). The majority of the sample were women (MD: 53.33%; CG1: 53.12%), in an undergraduate course and with low income (Supplementary Table 1). The average patient had depression levels of mild severity (HAM-D: 12.56 ± 0.56). One patient had a comorbid anxiety disorder (Supplementary Figure 2). Patients showed worse sleep quality than controls and had similar BMI (Supplementary Table 1).

**FIGURE 1:**
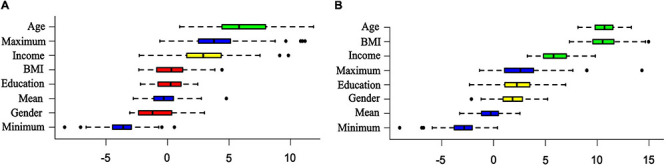
Importance of sociodemographic characteristics for discrimination of patients from controls. The random forest–based algorithm (Boruta test) of sociodemographic characteristics relevant to discrimination of: **(A)** patients with first episode of major depression (MD, *n* = 30) and the control group (CG1, *n* = 32). **(B)** Patients with treatment-resistant major depression (TRD, *n* = 28) and the respective control group (CG2, *n* = 30). The colors used in the images denote: green, relevant characteristic; yellow, tentative of relevance; red, no relevant characteristic; and blue, randomly shuffled data at a maximum, mean, and minimum levels.

Among the sociodemographic characteristics, the Boruta algorithm showed that only age (*I* = 6.03) was relevant for discrimination between MD and CG1 groups (Figure 1A).

**FIGURE 2:**
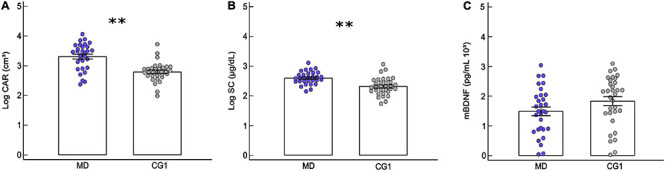
Comparison of physiological parameters between *de novo* patients and its respective control group. Mean ± standard error of (MANCOVA test): **(A)** salivary cortisol awakening response (CAR) of patients with first episode of major depression (MD: *n* = 30) and healthy controls (CG1: *n* = 32), **(B)** serum cortisol (SC) levels for MD and CG1, and **(C)** serum mature brain-derived neurotrophic factor (mBDNF) for MD and CG1. ^∗∗^*p* ≤ 0.001. The circles correspond to the data of each volunteer.

A significant statistical effect was found for the levels of SC, CAR, and mBDNF between groups (*F* = 7.56, *p* < 0.001) (Supplementary Table 2). Patients with first episode of MDD had higher salivary levels of CAR (*F* = 27.61, *p* < 0.001; *d* = 1.33, CI: 0.77 to 1.89) and SC (*F* = 19.44, *p* < 0.001; *d* = 1.08, CI: 0.54 to 1.63) than the control group (Figures 2A,B and Supplementary Tables 2, 3). Moreover, MD and CG1 had similar levels of mBDNF (*F* = 2.73, *p* = 0.10; *d* = −0.42, CI: −0.94 to −0.09) (Figure 2C and Supplementary Tables 2, 3). Age was controlled in these analyses, but it was revealed as a relevant covariate for only SC (*F* = 5.03, *p* = 0.02) (Supplementary Table 2), where the levels of SC decreased with the age of volunteer (Spearman’s test: *rho* = −0.28, *p* = 0.02). The majority of the MD group (96.6%) and all CG1 (100%) did not reveal sufficient levels of CRP to enable a positive result in the qualitative test (thus, denoting a potentially low level of inflammation). Therefore, the subsequent quantitative CRP analysis was not completed for these groups.

#### Patients With Treatment-Resistant Depression

Patients with TRD (*n* = 28) and their respective control group (CG2: *n* = 30) were Brazilian adults (TRD: 41.57 ± 11.61; CG2: 32.34 ± 1.95), with the majority being women (TRD: 75%; CG2: 76.67%). The patient group had a larger proportion of volunteers with lower income and education than controls (Supplementary Table 1). The TRD sample on average had severe-level symptoms of MD (HAM-D: 21.57 ± 0.99), with approximately 10 years of MDD and an average of 12 episodes. Twenty (71.42%) patients with TRD had a personality disorder (histrionic: *n* = 10/50%; borderline: *n* = 9/45%; schizoid: *n* = 1/5%) and 12 (42.85%) had anxiety disorder (generalized anxiety disorder: *n* = 10/83.33%; panic disorder: *n* = 5/17.24; social phobia: *n* = 2/16.67%). It may be noticed that some patients could show more than one comorbidity (Supplementary Figure 2). Patients with TRD had worse sleep quality and higher BMI than the control group (Supplementary Table 1). A large proportion of the patients (57.1%) had tried two to three unsuccessful earlier treatments with different antidepressants, 28.6% tested four to five, and 14.3% tested six to nine. All patients were previously treated with a selective serotonin reuptake inhibitor. Tricyclic antidepressants were used as the second most common treatment (Supplementary Figure 3).

**FIGURE 3:**
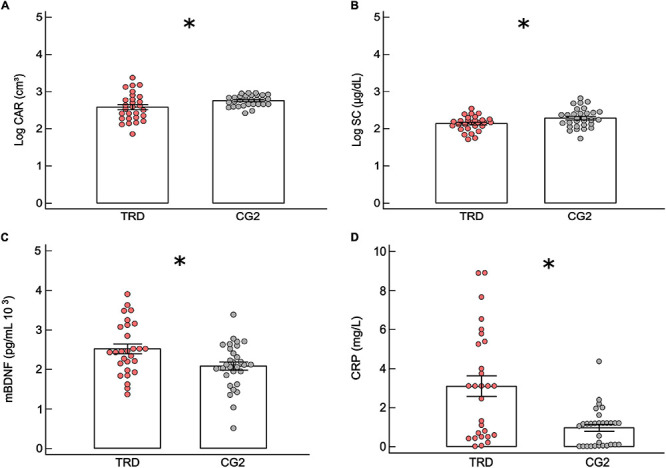
Comparison of physiological parameters between treatment-resistant and its respective control group. Mean ± standard error of (MANCOVA test): **(A)** cortisol awakening response (CAR) for patients with treatment-resistant major depression (TRD: *n* = 28) and control group (CG2: *n* = 30). **(B)** Serum cortisol (SC) levels for TRD and CG2. **(C)** Serum mature brain-derived neurotrophic factor (mBDNF) for TRD and CG2. **(D)** Mean ± standard error of (GLM test) serum C-reactive protein (CRP) for TRD and CG2. The circles are the data of volunteers. ^∗^*p* ≤ 0.05.

Age (*I* = 10.64), BMI (*I* = 10.56), and income (*I* = 5.94) were relevant for discrimination of TRD and CG2 groups (Figure 1B). There was a significant multivariate effect between SC, CAR, and mBDNF (*F* = 3.32, *p* = 0.008). Patients with TRD presented lower levels of CAR (group: *F* = 5.39, *p* = 0.02; *d* = −0.62, CI: −1.16 to −0.08) and SC than controls (group: *F* = 6.27, *p* = 0.01; BMI: *F* = 6.05, *p* = 0.01; *d* = −0.64 CI: −1.18 to −0.10) (Figure 3A,B and Supplementary Tables 2, 3), where the level of SC was controlled by BMI (*F* = 6.05, *p* = 0.01; Spearman’s test: *rho* = 0.14, *p* = 0.06). Moreover, patients with TRD presented higher levels of mBDNF than controls (group: *F* = 7.58, *p* = 0.008; *d* = 0.70, CI: 0.16 to 1.24), which was controlled for income (*F* = 4.40, *p* = 0.04) (Figure 3C and Supplementary Tables 2, 3), where the levels of mBDNF revealed a trend toward being higher in the lower-income volunteers (*t* = 1.59, *p* = 0.05). The levels of CRP were also higher in patients with TRD than in healthy volunteers (GLM; group: *F* = 5.11, *p* = 0.02; *d* = 1.05 CI: 0.48 to 1.61) (Figure 3D and Supplementary Tables 2, 3), which was controlled for BMI (*F* = 14.65, *p* < 0.001), the levels of CRP increased with volunteer BMI (Spearman’s test: *rho* = 0.47, *p* < 0.001).

### Physiological Changes and Depression Severity

The selection of HAM-D sub-domains by the Boruta algorithm showed that H11 (*I* = 10.95), H15 (*I* = 10.15), H1 (*I* = 9.53), H12 (*I* = 9.24), H7 (*I* = 9.21), H8 (*I* = 5.58), H9 (*I* = 4.56), and H14 (*I* = 4.24) were relevant for discrimination between MD and TRD groups (Supplementary Figure 3). Therefore, sHAM-D was calculated for each patient using the sum of these relevant selected sub-domains (sHAM-D: MD = 5.43 ± 0.29, TRD = 12.03 ± 0.61) (Supplementary Figure 4).

The selection of PSQI sub-domains by the Boruta algorithm showed that C6 (*I* = 36.46) and C4 (*I* = 4.93) were relevant for discrimination between MD and TRD groups (Supplementary Figure 4), and the sub-score calculated (sPSQI: MD = 0.90 ± 0.23, TRD = 4.14 ± 0.32).

**FIGURE 4:**
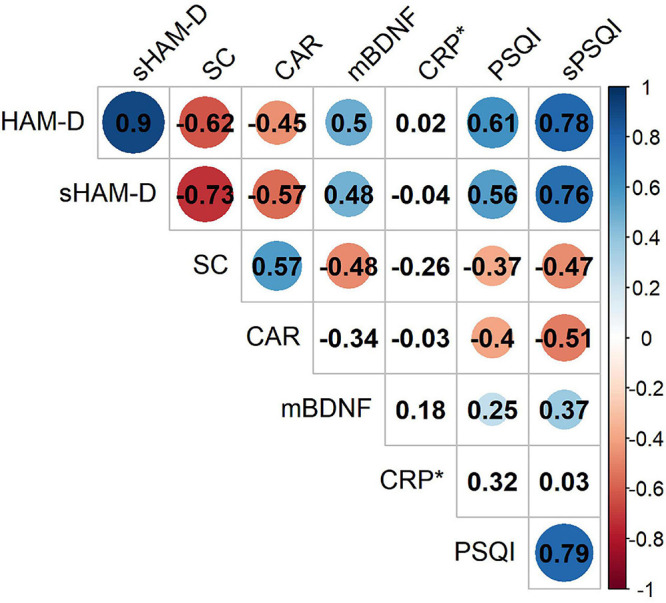
Spearman’s correlation matrix of physiological parameters across depression severity (measured by HAM-D and sHAM-D) and sleep quality (measured by PSQI and sPSQI) for both patient groups (i.e., MD and TRD) gathering (*n* = 58). Circles denote significant correlations. The area and color of the circles represent the strength and direction (red, negative and blue, positive) of the correlation, respectively. *p-values for CRP were calculated considering only patients with treatment-resistant major depression.

The levels of SC and the CAR decrease with depression symptom severity, assessed by both HAM-D (SC: *rho* = −0.62, *p* < 0.001; CAR: *rho* = −0.45, *p* = 0.001) and sHAM-D (SC: *rho* = −0.73, *p* < 0.001; CAR: *rho* = −0.57, *p* < 0.001) (Figure 4 and Supplementary Figure 5). Again, the levels of SC (Spearman’s test; PSQI: *rho* = −0.37, *p* = 0.004; sPSQI: *rho* = −0.47, *p* < 0.001) and CAR (PSQI: *rho* = −0.4, *p* = 0.002; sPSQI: *rho* = −0.51, *p* < 0.001) decreased with worse sleep quality (i.e., high score and sub-score in PSQI) (Figure 4). Conversely, the level of mBDNF increased with symptom severity, measured by HAM-D (*rho* = 0.50, *p* < 0.001) and sHAM-D (*rho* = 0.48, *p* < 0.001), and with worse sleep quality (PSQI: *rho* = 0.25, *p* = 0.05; sPSQI: *rho* = 0.37, *p* = 0.001) (Figure 4 and Supplementary Figure 5). We did not find significant correlations for CRP (Figure 4 and Supplementary Figure 5).

Earlier use of antidepressants predicted lower CAR (β = −1.32, *t* = −6.69, *p* < 0.001, *R*^2^ = 0.44) and SC (β = −1.53, *t* = −9.07, *p* < 0.001, *R*^2^ = 0.59) and higher mBDNF levels (β = 1.15, *t* = 5.36, *p* < 0.001, *R*^2^ = 0.33). However, the number of treatments did not modulate any of those factors (CAR: *rho* −0.13 *p* = 0.48; SC: *rho* = 0.04, *p* = 0.83; mBDNF: *rho* = −0.22, *p* = 0.25; CRP: *rho* = −0.16, *p* = 0.40).

## Discussion

As per our hypothesis, the results of our present study reveal that patients with MDD present with differing cortisol, mBDNF, and CRP profiles than that observed in healthy volunteers. Furthermore, these molecular changes were highly dependent on the stages of MDD, i.e., the severity of depressive symptoms and the sleep quality.

The levels of both serum and saliva (i.e., CAR) cortisol were higher in the patients with *de novo* MDD, whereas it was presented in lower levels in the TRD group compared with the respective healthy controls. Moreover, moderate correlations were found between the lower levels of both cortisol measures and stronger depressive symptoms. In the literature, some studies have revealed that hypercortisolemia and higher CAR are usually observed in patients with the short-term depression (Foreman and Goodyer, 1988; Lopez-Duran et al., 2009; Dedovic and Ngiam, 2015), whereas other specify that patients with TRD and with a long-term disease have hypocortisolemia and a blunted CAR (Stetler and Miller, 2005; Vreeburg et al., 2013; Moreira et al., 2016; Bremmer et al., 2017; Galvão et al., 2018). In contrast, studies that analyze changes in cortisol over clinical progression of MDD are sporadic, but like our findings, they support the notion of reduction in cortisol levels over disease severity (Verduijn et al., 2015).

One of the main risk factors for MDD is chronic stress (Mazure, 1998; Dohrenwend, 2006); although the reactivity of the HPA axis to a stressor is an adaptive function, the absence of its recovery induces many dysfunctions (Sen et al., 2008; Sousa et al., 2015). The sustained overstimulation of the HPA axis, caused by an impaired negative feedback from the HPA axis, results in hypercortisolemia (Dean and Keshavan, 2017), as it is found in patients with mild and moderate MDD (Gold et al., 1988; Juruena and Cleare, 2007; Dean and Keshavan, 2017; Ferrari and Villa, 2017), as well as in patients with Cushing’s syndrome (Gold et al., 1986), which share some similar symptoms such as metabolic syndrome (Pivonello et al., 2015). However, if the HPA axis function has been hyperactivated for a long time, this can later result in adrenal failure (Yehuda et al., 2005) or an increase in the sensitivity of cortisol negative feedback in the hypothalamus (Sriram et al., 2012), thus switching such profile from hypercortisolemia to hypocortisolemia, as it is seen in patients with TRD (Herbert, 2013) and in individuals with Addison’s syndrome who show chronic fatigue syndrome (Maripuu et al., 2014). Therefore, our findings importantly reveal the contrast in cortisol changes between *de novo* and TRD depression, suggesting a switch from hypercortisolemia to hypocortisolemia as the MDD gets worse and longer, thus helping for better understanding about HPA changes in MDD (Vreeburg et al., 2013; Bremmer et al., 2017; Galvão et al., 2018). This further supports the theory that these changes are related to the severity of depressive symptoms (Stetler and Miller, 2005; Karlović et al., 2012; Herbert, 2013).

We also found that the earlier use of antidepressants predicted lower levels of both cortisol measures (i.e., SC and CAR). Distinct classes of antidepressants may have opposite effects in the modulation of cortisol, which also depends on the treatment duration. Therefore, since patients with TRD in our study used many different classes of antidepressants and had distinct treatment schemes (dose and duration), it is difficult to assure wheter the low levels of cortisol found here are an etiology of the disorder or a result of earlier antidepressant treatments. In the case of *de novo* patients (who were naïve these medications), it may be speculated that their high levels of cortisol are associated with etiologic changes of MDD.

Therefore, these distinct cortisol changes in TRD and *de novo* patients should be taken into account in the choice of the antidepressant treatment, since cortisol is a pleiotropic hormone (Schüle, 2007; Galvão-Coelho et al., 2012). Mainly because, patients in remission who have ongoing HPA-axis dysfunction have higher relapse risk (Slavich and Irwin, 2014).

Furthermore, we found that patients with TRD had higher levels of mBDNF than healthy volunteers, while patients with first episode showed similar levels of mBDNF as compared to its control group. Indeed, a positive correlation between mBDNF levels and MDD severity was found. The increased mBDNF of patients with TRD are in opposition to our hypothesis. The neurodegenerative hypothesis of MDD and some studies in this field show low blood levels of BDNF in patients with MDD (Mannari et al., 2008; Monteleone et al., 2008; Lima-Ojeda et al., 2018), although this finding is not unanimous (Almeida et al., 2019; Verduijn et al., 2015; Vu and Student, 2009). Here, we found that earlier antidepressant treatment predicts high levels of mBDNF. Therefore, supported by earlier evidences (Pilar-Cuéllar et al., 2012; Zhou et al., 2017) and by observation that patients with first episode MDD, who were naïve for antidepressant medication, presented similar levels of mBDNF of the control group, we might speculate that the increased levels of mBDNF are potentially due to the long-term use of antidepressants by patients with TRD (Perito and Fortunato, 2012; Yoshida et al., 2012; Lima-Ojeda et al., 2018).

In addition, BDNF isoforms should be taken into account. Most studies in patients with MDD analyzed the levels of total blood BDNF (Vu and Student, 2009; Shirayama et al., 2015; Almeida et al., 2019); however, we measured its active isoform, i.e., the mBDNF. The few studies that examined mBDNF in patients with MDD have showed both low and high levels of this isoform when compared with controls (Foltran and Diaz, 2016; Angoa-Perez et al., 2017). Importantly, it is proposed that the pro-BDNF/mature BDNF (mBDNF) ratio should be more relevant for shaping physiological or pathological conditions (Raison et al., 2013; Zhao et al., 2017; Mora et al., 2018; Osimo et al., 2019).

The concentration of CRP was not measured for patients with first MDD episode since 96.6% did not show measurable levels of CRP and, thereby, a absence of inflammation. Earlier, other studies in drug-naïve patients with MDD or in patients with initial stages of this disorder also did not detect increases in CRP or IL-6 compared with controls (Cubala and Landowski, 2014; Verduijn et al., 2015; Zou et al., 2018).

However, patients with TRD had higher levels of CRP than healthy volunteers, which was characterized as a low-grade inflammatory profile (CRP level >3 mg/L; healthy adult population <1 mg/L) (Angoa-Perez et al., 2017; Zhao et al., 2017). Some studies have identified CRP as an etiological factor for MDD (Harris et al., 1999; Vreeburg et al., 2013; Köhler-Forsberg et al., 2016) and pointed its increase regardless of IL-6 (Ivashchenko et al., 2005; Saito et al., 2016). Since the measurement of CRP shows some advantages, such as high stability and low-cost dosage, it is proposed that CRP could be used alone as an inflammatory biomarker in MDD, independently of IL-6 (Carboni, 2013; Galvão-Coelho et al., 2020). A significant correlation between CRP and MDD severity is seen in some studies (Carboni, 2013; Köhler-Forsberg et al., 2016), although it is not unanimous (Verduijn et al., 2015). Here, we did not find this correlation possibly due to our small sample size for CRP quantitative data. Although we have found between-group differences among these physiological parameters, it is important to note that these inflammatory changes are not exclusive of depression and may occur in other diseases. Therefore, the changes in these physiological parameters cannot be exclusive biomarkers of MDD (Mondelli et al., 2010; Martinotti et al., 2016; Orsolini et al., 2018).

Moreover, we must highlight that the physiological changes observed in our patients remained significant even after controlling for relevant individual and social characteristics. For the patients with first episode and their control, a weak inverse correlation was found between the levels of SC and age, although the age dispersion is small in this sample. Some studies have showed age-related changes of the HPA axis in healthy and clinical populations (Yiallouris et al., 2019). For patients with TRD and their control, we found a positive correlation between BMI and the levels of cortisol and CRP. High levels of proinflammatory biomarkers can be observed in individuals with overweight due to an overactive metabolism of cortisone into cortisol in the adipose tissue and also due to a local immune activity (Milaneschi et al., 2019). Moreover, the lower-income volunteers had higher levels of mBDNF, probably a consequence of earlier antidepressant treatment, since low-income is expected to be a strong stressor, what would lower mBDNF levels (Fung et al., 2015). Therefore, these results show the importance of controlling potential sociodemographic modulators of physiological parameters, avoiding erroneous interpretations.

We observed that worse sleep quality is related to lower levels of both cortisol parameters. A blunted CAR has been observed in patients with MDD and sleep disorders (Santiago et al., 2020). Patients with MDD have presented changes in slow wave sleep as a result of reduction in HPA axis activity (Balbo et al., 2010). Currently, there is no clear conclusion about the relationship between cortisol and sleep changes, whether this hormonal change is a cause or a consequence of sleep disturbances (Steiger, 2003).

Finally, worse sleep quality was weakly correlated with higher levels of mBDNF. Despite some studies showing that high levels of BDNF are related to a good sleep profile (Giese et al., 2014), MDD patients with sleep disorders did not show low levels of serum BDNF (Santiago et al., 2020). Interestingly, therapies using acute sleep deprivation for MDD increased mBDNF levels (Schmitt et al., 2016). Moreover it can be noted that, although sleep changes are potentially in part related to inflammation (Patel et al., 2009; Chrousos et al., 2016), we did not find correlations between these elements, which can be in part due to the reduced sample size of patients who had their quantitative value of CRP measured.

Recently, some studies have explored whether various biomarkers are related to the specific symptoms of the psychopathologies, rather than to the global condition (Yoshida et al., 2012; Caldieraro et al., 2017). For instance, the HAM-D sub-domains have been used to assess the response to antidepressants (Silverstone et al., 2002). In our study, the HAM-D sub-score, which was the sum of HAM-D sub-domains selected by the Boruta algorithm, had a weak contribution to the improvement of the correlation strength between depression severity and physiological parameters. Similar founds were observed for PSQI sub-scores.

It is essential to point out some limitations of this study, such as the modest number of volunteers, the cross-sectional design with a single sample of blood and saliva per subject, and the lack of pro-BDNF measurement, which is relevant to evaluate the relationship between m-BDNF and pro-BDNF. It is also important to highlight that some psychometric instruments were filled out by the clinician in contrast to others of self-report. Therefore, studies with larger populations of patients with MDD and designed with sequential biological measurements throughout the day and on different days are encouraged. Moreover, it is of important to evidence that only around 30% of depressive patients show a progression to TRD (Demyttenaere and Van Duppen, 2019), although it is believed that understanding the physiological changes in this group is important to give pavement to studies of MDD biomarkers and new treatments.

In conclusion, these results show that changes in some physiological parameters are distinct according to the clinical stage of depression. This understanding would be important to support the precision psychiatry, to stimulate further studies on biological assessments for depression, and to investigate how it may be underlying the lack of clinical response to usual pharmacotherapies.

## Data Availability Statement

The datasets presented in this article are not readily available because the sharing of this data set was not previously authorized by the local ethics committee. Requests to access the datasets should be directed to NG-C, nicolelgalvaocoelho@gmail.com.

## Ethics Statement

The studies involving human participants were reviewed and approved by the Onofre Lopes University Hospital (HUOL) Medical Research Ethics Committee (# 579,479) and Federal University of Rio Grande do Norte (UFRN) Human Research Ethics Committee (# 2.628.202). The patients/participants provided their written informed consent to participate in this study.

## Author Contributions

NG-C, DA, FP-F, BL-S, JM-d-O, EN, and ML-M planned the clinical trial. FP-F and DA screened volunteers. AM and RA measured the hormonal data. AM and GS carried out statistical analysis and illustrated the figures and tables. All authors contributed to the manuscript.

## Conflict of Interest

The authors declare that the research was conducted in the absence of any commercial or financial relationships that could be construed as a potential conflict of interest.

## Publisher’s Note

All claims expressed in this article are solely those of the authors and do not necessarily represent those of their affiliated organizations, or those of the publisher, the editors and the reviewers. Any product that may be evaluated in this article, or claim that may be made by its manufacturer, is not guaranteed or endorsed by the publisher.

## References

[B1] Almeida R. N. Galvão A. C. M. Da Silva F. S. Silva E. A. D. S. Palhano-Fontes F. Maia-de-Oliveira J. P. (2019). Modulation of serum brain-derived neurotrophic factor by a single dose of ayahuasca: observation from a randomized controlled trial. *Front. Psychol.* 10:1234. 10.3389/fpsyg.2019.01234 31231276 PMC6558429

[B2] American Psychiatric Association (2013). *Diagnostic and Statistical Manual of Mental Disorders (Fifth ed.).* Arlington: American Psychiatric Publishing.

[B3] Angoa-Perez M. Anneken J. H. Kuhn D. M. (2017). The role of brain-derived neurotrophic factor in the pathophysiology of psychiatric and neurological disorders. *J. Psychiatry Psychiatr. Disord.* 1:1026502. 10.26502/jppd.2572-519X0024 PMC8793768 35098038

[B4] Balbo M. Leproult R. Van Cauter E. (2010). Impact of sleep and its disturbances on hypothalamo-pituitary-adrenal axis activity. *Int. J. Endocrinol.* 2010:759234. 10.1155/2010/759234 20628523 PMC2902103

[B5] Bertolazi A. N. Fagondes S. C. Hoff L. S. Dartora E. G. da Silva Miozzo I. C. de Barba M. E. F. (2011). Validation of the Brazilian Portuguese version of the Pittsburgh sleep quality index. *Sleep Med.* 12 70–75. 10.1016/j.sleep.2010.04.020 21145786

[B6] Bremmer M. A. Deeg D. J. Beekman A. T. Penninx B. W. Lips P. Hoogendijk W. J. (2017). Major Depression in Late Life Is Associated with Both Hypo- and Hypercortisolemia. *Biol. Psychiatry* 62 479–486. 10.1016/j.biopsych.2006.11.033 17481591

[B7] Breslau N. Roth T. Rosenthal L. Andreski P. (1996). Sleep disturbance and psychiatric disorders: a longitudinal epidemiological study of young adults. *Biol. Psychiatry* 39 411–418. 10.1016/0006-3223(95)00188-3 8679786

[B8] Buysse D. J. Reynolds I. I. I. C. F. Monk T. H. Berman S. R. Kupfer D. J. (1989). The Pittsburgh Sleep Quality Index: a new instrument for psychiatric practice and research. *Psychiatry Res.* 28 193–213. 10.1016/0165-1781(89)90047-4 2748771

[B9] Caldieraro M. A. Vares E. A. Souza L. H. Spanemberg L. Guerra T. A. Wollenhaupt-Aguiar B. (2017). Illness severity and biomarkers in depression: using a unidimensional rating scale to examine BDNF. *Compr. Psychiatry* 75 46–52. 10.1016/j.comppsych.2017.02.014 28301802

[B10] Carboni L. (2013). Peripheral biomarkers in animal models of major depressive disorder. *Dis. Markers* 35:33. 10.1155/2013/284543 24167347 PMC3774958

[B11] Chida Y. Steptoe A. (2009). Cortisol awakening response and psychosocial factors: a systematic review and meta-analysis. *Biol. Psychol.* 80 265–278. 10.1016/j.biopsycho.2008.10.004 19022335

[B12] Chrousos G. Vgontzas A. N. Kritikou I. (2016). “HPA axis and sleep,” in *Endotext [Internet]*, eds Feingold K. R. Anawalt B. Boyce A. (South Dartmouth: MDText.com, Inc).

[B13] Conway C. R. George M. S. Sackeim H. A. (2017). Toward an evidence-based, operational definition of treatment-resistant depression: when enough is enough. *JAMA Psychiatry* 74 9–10. 10.1001/jamapsychiatry.2016.2586 27784055

[B14] Cubala W. J. Landowski J. C. - (2014). reactive protein and cortisol in drug-naïve patients with short-illness-duration first episode major depressive disorder: possible role of cortisol immunomodulatory action at early stage of the disease. *J. Affect. Disord.* 152 534–537. 10.1016/j.jad.2013.10.004 24161452

[B15] Dean J. Keshavan M. (2017). The neurobiology of depression: an integrated view. *Asian J. Psychiatr.* 27 101–111. 10.1016/j.ajp.2017.01.025 28558878

[B16] Dedovic K. Ngiam J. (2015). The cortisol awakening response and major depression: examining the evidence. *Neuropsychiatr. Dis. Treat.* 11 1181–1189. 25999722 10.2147/NDT.S62289 PMC4437603

[B17] Demyttenaere K. Van Duppen Z. (2019). The impact of (the concept of) treatment-resistant depression: an opinion review. *Int. J. Neuropsychopharmacol.* 22 85–92. 10.1093/ijnp/pyy052 29961822 PMC6368367

[B18] Dohrenwend B. P. (2006). Inventorying stressful life events as risk factors for psychopathology: toward resolution of the problem of intracategory variability. *Psychol. Bull.* 132 477–495. 10.1037/0033-2909.132.3.477 16719570 PMC1584216

[B19] Elfeddali I. der Feltz-Cornelis V. Christina M. Van Os J. Knappe S. Vieta E. (2014). Horizon 2020 priorities in clinical mental health research: results of a consensus-based ROAMER expert survey. *Int. J. Environ. Res. Public Health* 11 10915–10939. 10.3390/ijerph111010915 25337940 PMC4211014

[B20] Faul F. Erdfelder E. Buchner A. Lang A.-G. (2009). Statistical power analyses using G^∗^Power 3.1: tests for correlation and regression analyses. *Behav. Res. Methods* 41 1149–1160. 10.3758/BRM.41.4.1149 19897823

[B21] Ferrari F. Villa R. F. (2017). The neurobiology of depression: an integrated overview from biological theories to clinical evidence. *Mol. Neurobiol.* 54 4847–4865. 10.1007/s12035-016-0032-y 27510505

[B22] Foltran R. B. Diaz S. L. B. D. N. F. (2016). isoforms: a round trip ticket between neurogenesis and serotonina?. *J. Neurochem.* 138 204–221. 10.1111/jnc.13658 27167299

[B23] Foreman D. M. Goodyer I. M. (1988). Salivary cortisol hypersecretion in juvenile depression. *J. Child Psychol. Psychiatry* 29 311–320. 10.1111/j.1469.7610.1988.tb00719.x 3417807

[B24] Fried E. I. von Stockert S. Haslbeck J. M. B. Lamers F. Schoevers R. A. Penninx B. W. J. H. (2019). Using network analysis to examine links between individual depressive symptoms, inflammatory markers, and covariates. *Psychol. Med.* 50 2682–2690. 10.1017/S0033291719002770 31615595

[B25] Fung J. Gelaye B. Zhong Q. Y. Rondon M. B. Sanchez S. E. Barrios Y. V. (2015). Association of decreased serum brain-derived neurotrophic factor (BDNF) concentrations in early pregnancy with antepartum depression. *BMC Psychiatry* 15:43. 10.1186/s12888-015-0428-7 25886523 PMC4364091

[B26] Galvão A. C. M. de Almeida R. N. Silva E. A. Freire F. A. Palhano-Fontes F. Onias H. (2018). Cortisol modulation by ayahuasca in patients with treatment resistant depression and healthy controls. *Front. Psychiatry* 9:185. 10.3389/fpsyt.2018.00185 29867608 PMC5952178

[B27] Galvão-Coelho N. L. de Menezes Galvão A. C. de Almeida R. N. Palhano-Fontes F. Campos Braga I. Lobão Soares B. (2020). Changes in inflammatory biomarkers are related to the antidepressant effects of Ayahuasca. *J. Psychopharmacol.* 34 1125–1133. 10.1177/0269881120936486 32648790

[B28] Galvão-Coelho N. L. Silva H. P. A. Sousa M. B. C. (2012). Response to stress: II. Resilience and vulnerability. *Estudos de Psicologia* 20 72–81. 10.5935/1678-4669.20150009

[B29] Giese M. Unternährer E. Hüttig H. Beck J. Brand S. Calabrese P. (2014). BDNF: an indicator of insomnia?. *Mol. Psychiatry* 19 151–152. 10.1038/mp.2013.10 23399916 PMC3903111

[B30] Gold P. W. Goodwin F. K. Chrousos G. P. (1988). Clinical and biochemical manifestations of depression. *N. Engl. J. Med.* 319 413–420. 10.1155/2015/581976 3041279

[B31] Gold P. W. Loriaux D. L. Roy A. Kling M. A. Calabrese J. R. Kellner C. H. (1986). Responses to corticotropin-releasing hormone in the hypercortisolism of depression and Cushing’s disease. *N. Engl. J. Med.* 314 1329–1335. 10.1056/NEJM198605223142101 3010108

[B32] Groves J. O. (2007). Is it time to reassess the BDNF hypothesis of depression?. *Mol. Psychiatry* 12 1076–1088. 10.1038/sj.mp.4002075 17700574

[B33] Haapakoski R. Mathieu J. Ebmeier K. P. Alenius H. Kivimäki M. (2015). Cumulative meta-analysis of interleukins 6 and 1β, tumour necrosis factor α and C-reactive protein in patients with major depressive disorder. *Brain Behav. Immun.* 49 206–215. 10.1016/j.bbi.2015.06.001 26065825 PMC4566946

[B34] Halfaker D. A. Akeson S. T. Hathcock D. R. Mattson C. Wunderlich T. L. (2011). “Psychological Aspects of Pain,” in *Pain Procedures in Clinical Practice*, eds Lennard T. A. Walkowski S. Singla A. K. Vivian D. G. (New York: Elsevier).

[B35] Hamilton M. A. (1960). Rating Scale for Depression. *J. Neurol. Neurosurg. Psychiatry* 23 56–62. 10.1136/jnnp.23.1.56 14399272 PMC495331

[B36] Harris T. B. Ferrucci L. Tracy R. P. Corti M. C. Wacholder S. Ettinger W. H. Jr. (1999). Associations of elevated interleukin-6 and C-reactive protein levels with mortality in the elderly. *Am. J. Med.* 106 506–512. 10.1016/s0002-9343(99)00066-2 10335721

[B37] Hashimoto K. (2015). Brain-derived neurotrophic factor (BDNF) and its precursor proBDNF as diagnostic biomarkers for major depressive disorder and bipolar disorder. *Eur. Arch. Psychiatry Clin. Neurosci.* 265 83–84. 10.1007/s00406-014-0557-x 25362578

[B38] Herbert J. (2013). Cortisol and depression: three questions for psychiatry. *Psychol. Med.* 43 449–469. 10.1017/S0033291712000955 22564216

[B39] Howland R. (2008). Sequenced Treatment Alternatives to Relieve Depression (STAR^∗^D). *J. Psychosoc. Nurs. Ment. Health Serv.* 46 21–24. 10.3928/02793695-20081001-05 18822996

[B40] Insel T. Cuthbert B. Garvey M. Heinssen R. Pine D. S. Quinn K. (2010). Research domain criteria (RDoC): toward a new classification framework for research on mental disorders. *Am. J. Psychiatry* 167 748–751. 10.1176/appi.ajp.2010.09091379 20595427

[B41] Ivashchenko Y. Krammer F. Schäfer S. Bucher A. Veit K. Hombach V. (2005). Protein kinase C pathway is involved in transcriptional regulation of C-reactive protein synthesis in human hepatocytes. *Arterioscler. Thromb. Vasc. Biol.* 25 186–192. 10.1161/01.ATV.0000150041.81963.68 15539624

[B42] Juruena M. F. Cleare A. J. (2007). Overlap between atypical depression, seasonal affective disorder and chronic fatigue syndrome. *Braz. J. Psychiatry* 29 S19–S26. 10.1590/S1516-44462007000500005 17546343

[B43] Karege F. Perret G. Bondolfi G. Schwald M. Bertschy G. Aubry J. M. (2002). Decreased serum brain-derived neurotrophic factor levels in major depressed patients. *Psychiatry Res.* 109 143–148. 10.1016/s0165-1781(02)00005-7 11927139

[B44] Karlović D. Serretti A. Vrkić N. Martinac M. Marčinko D. (2012). Serum concentrations of CRP, IL-6, TNF-α and cortisol in major depressive disorder with melancholic or atypical features. *Psychiatry Res.* 198 74–80. 10.1016/j.psychres.2011.12.007 22386567

[B45] Kim Y. K. (2020). Major Depressive Disorder: current Advances and Paradigm Shifts. *Psychiatry Investig.* 17 179–180. 10.30773/pi.2019.0092 32209964 PMC7113174

[B46] Köhler-Forsberg O. Krogh J. Mors O. Eriksen Benros M. (2016). Inflammation in depression and the potential for anti-inflammatory treatment. *Curr. Neuropharmacol.* 14 732–742. 10.2174/1570159X14666151208113700 27640518 PMC5050394

[B47] Koo P. C. Berger C. Kronenberg G. Bartz J. Wybitul P. Reis O. (2019). Combined cognitive, psychomotor and electrophysiological biomarkers in major depressive disorder. *Eur. Arch. Psychiatry Clin. Neurosci.* 269 823–832. 10.1007/s00406-018-0952-9 30392042

[B48] Krishnadas R. Cavanagh J. (2012). Depression: an inflammatory illness? *J. Neurol. Neurosurg. Psychiatry* 83 495–502. 10.1136/jnnp-2011-301779 22423117

[B49] Kunugi H. Hori H. Ogawa S. (2015). Biochemical markers subtyping major depressive disorder. *Psychiatry Clin. Neurosci.* 69 597–608. 10.1111/pcn.12299 25825158

[B50] Kursa M. B. Rudnicki W. R. (2010). Feature selection with the boruta package. *J. Stat. Softw.* 36 1–13. 10.18637/jss.v036.i11

[B51] Lee B. H. Kim Y. K. (2009). Reduced platelet BDNF level in patients with major depression. *Prog. Neuropsychopharmacol. Biol. Psychiatry* 33 849–853. 10.1016/j.pnpbp.2009.04.002 19371767

[B52] Lima-Ojeda J. M. Rupprecht R. Baghai T. C. (2018). Neurobiology of depression: a neurodevelopmental approach. *World J. Biol. Psychiatry* 19 349–359. 10.1080/15622975.2017.1289240 28155577

[B53] Liu Q. He H. Yang J. Feng X. Zhao F. Lyu J. (2020). Changes in the global burden of depression from 1990 to 2017: findings from the global burden of disease study. *J. Psychiatr. Res.* 126 134–140. 10.1016/j.jpsychires.2019.08.002 31439359

[B54] Lopez-Duran N. L. Kovacs M. George C. J. (2009). Hypothalamic–pituitary–adrenal axis dysregulation in depressed children and adolescents: a meta-analysis. *Psychoneuroendocrinology* 34 1272–1283. 10.1016/j.psyneuen.2009.03.016 19406581 PMC2796553

[B55] Lopresti A. L. Maker G. L. Hood S. D. Drummond P. D. A. (2014). review of peripheral biomarkers in major depression: the potential of inflammatory and oxidative stress biomarkers. *Prog. Neurpsychopharmacol. Biol. Psychiatry* 48 102–111. 10.1016/j.pnpbp.2013.09.017 24104186

[B56] Mannari C. Origlia N. Scatena A. Del Debbio A. Catena M. Dell’Agnello G. (2008). BDNF level in the rat prefrontal cortex increases following chronic but not acute treatment with duloxetine, a dual acting inhibitor of noradrenaline and serotonin re-uptake. *Cell. Mol. Neurobiol.* 28 457–468. 10.1007/s10571-007-9254-x 18172756 PMC11515045

[B57] Maripuu M. Wikgren M. Karling P. Adolfsson R. Norrback K. F. (2014). Relative hypo-and hypercortisolism are both associated with depression and lower quality of life in bipolar disorder: a cross-sectional study. *PLoS One* 9:e98682. 10.1371/journal.pone.0098682 24932586 PMC4059634

[B58] Marques A. H. Silverman M. N. Sternberg E. M. (2010). Evaluation of stress systems by applying noninvasive methodologies: measurements of neuroimmune biomarkers in the sweat, heart rate variability and salivary cortisol. *Neuroimmunomodulation* 17 205–208. 10.1159/000258725 20134204 PMC2917732

[B59] Martinotti G. Pettorruso M. De Berardis D. Varasano P. A. Lucidi Pressanti G. De Remigis V. (2016). Agomelatine Increases BDNF Serum Levels in Depressed Patients in Correlation with the Improvement of Depressive Symptoms. *Int. J. Neuropsychopharmacol.* 19:yw003. 10.1093/ijnp/pyw003 26775293 PMC4886672

[B60] Mazure C. M. (1998). Life stressors as risk factors in depression. *Clin. Psychol.* 5 291–313. 10.1111/j.1468-2850.1998.tb00151.x

[B61] Mello A. F. Juruena M. F. Pariante C. M. Tyrka A. R. Price L. H. Carpenter L. L. (2007). Depression and stress: is there an endophenotype? *Braz. J. Psychiatry* 29 S13–S18. 10.1590/s1516-44462007000500004 17546342 PMC4467732

[B62] Menke A. (2018). Precision pharmacotherapy: psychiatry’s future direction in preventing, diagnosing, and treating mental disorders. *Pharmacogenomics Pers. Med.* 11 211–222. 10.2147/PGPM.S146110 30510440 PMC6250105

[B63] Milaneschi Y. Simmons W. K. van Rossum E. F. C. Penninx B. W. (2019). Depression and obesity: evidence of shared biological mechanisms. *Mol. Psychiatry* 24 18–33. 10.1038/s41380-018-0017-5 29453413

[B64] Mondelli V. Dazzan P. Hepgul N. Di Forti M. Aas M. D’Albenzio A. (2010). Abnormal cortisol levels during the day and cortisol awakening response in first-episode psychosis: the role of stress and of antipsychotic treatment. *Schizophr. Res.* 116 234–242. 10.1016/j.schres.2009.08.013 19751968 PMC3513410

[B65] Monteleone P. Serritella C. Martiadis V. Maj M. (2008). Decreased levels of serum brain-derived neurotrophic factor in both depressed and euthymic patients with unipolar depression and in euthymic patients with bipolar I and II disorders. *Bipolar Disord.* 10 95–100. 10.1111/j.1399-5618.2008.00459.x 18199246

[B66] Moos R. H. Cronkite R. C. (1999). Symptom-Based Predictors of a 10-Year Chronic Course of Treated Depression. *J. Nerv. Ment. Dis.* 187 360–368. 10.1097/00005053-199906000-00005 10379723

[B67] Mora C. Zonca V. Riva M. A. Cattaneo A. (2018). Blood biomarkers and treatment response in major depression. *Expert Rev. Mol. Diagn.* 18 513–529. 10.1080/14737159.2018.1470927 29701114

[B68] Moreira M. A. Guerra R. O. Freire A. D. N. F. dos Santos Gomes C. Maciel A. C. C. (2016). Depressive symptomatology and cortisol concentrations in elderly community residents: a cross-sectional study. *Aging Clin. Exp. Res.* 28 131–137. 10.1007/s40520-015-0374-8 25986238

[B69] Ohayon M. M. Roth T. (2003). Place of chronic insomnia in the course of depressive and anxiety disorders. *J. Psychiatr. Res.* 37 9–15. 10.1016/S0022-3956(02)00052-3 12482465

[B70] Orsolini L. Sarchione F. Vellante F. Fornaro M. Matarazzo I. Martinotti G. (2018). Protein-C Reactive as Biomarker Predictor of Schizophrenia Phases of Illness? A Systematic Review. *Curr. Neuropharmacol.* 16 583–606. 10.2174/1570159X16666180119144538 29357805 PMC5997872

[B71] Osimo E. F. Baxter L. J. Lewis G. Jones P. B. Khandaker G. M. (2019). Prevalence of low-grade inflammation in depression: a systematic review and meta-analysis of CRP levels. *Psychol. Med.* 49 1958–1970. 10.1017/S0033291719001454 31258105 PMC6712955

[B72] Patel S. R. Zhu X. Storfer-Isser A. Mehra R. Jenny N. S. Tracy R. (2009). Sleep duration and biomarkers of inflammation. *Sleep* 32 200–204. 10.1093/sleep/32.2.200 19238807 PMC2635584

[B73] Perito M. E. S. Fortunato J. J. (2012). Marcadores biológicos da depressão: uma revisão sobre a expressão de fatores neurotróficos. *Revista Neurociência* 20 597–603. 10.34024/rnc.2012.v20.8235

[B74] Perlis M. L. Giles D. E. Buysse D. J. Tu X. Kupfer D. J. (1997). Self-reported sleep disturbance as a prodromal symptom in recurrent depression. *J. Affect. Disord.* 42 209–212. 10.1016/s0165-0327(96)01411-5 9105962

[B75] Perna G. Alciati A. Daccò S. Grassi M. Caldirola D. (2020). Personalized psychiatry and depression: the role of sociodemographic and clinical variables. *Psychiatry Investig.* 17 193–206. 10.30773/pi.2019.0289 32160691 PMC7113177

[B76] Pilar-Cuéllar F. Vidal R. Pazos A. (2012). Subchronic treatment with fluoxetine and ketanserin increases hippocampal brain-derived neurotrophic factor, β-catenin and antidepressant-like effects. *Br. J. Pharmacol.* 165 1046–1057. 10.1111/j.1476-5381.2011.01516.x 21627639 PMC3346247

[B77] Pivonello R. Simeoli C. De Martino M. C. Cozzolino A. De Leo M. Iacuaniello D. (2015). Neuropsychiatric disorders in Cushing’s syndrome. *Front. Neurosci.* 9:129. 10.3389/fnins.2015.00129 25941467 PMC4403344

[B78] Pruessner J. C. Kirschbaum C. Meinlschmid G. Hellhammer D. H. (2003). Two formulas for computation of the area under the curve represent measures of total hormone concentration versus time-dependent change. *Psychoneuroendocrinology* 28 916–931. 12892658 10.1016/s0306-4530(02)00108-7

[B79] Raison C. L. Rutherford R. E. Woolwine B. J. Shuo C. Schettler P. Drake D. F. (2013). A randomized controlled trial of the tumor necrosis factor antagonist infliximab for treatment-resistant depression: the role of baseline inflammatory biomarkers. *JAMA Psychiatry* 70 31–41. 10.1001/2013.jamapsychiatry.4 22945416 PMC4015348

[B80] Roddy D. W. Farrell C. Doolin K. Roman E. Tozzi L. Frodl T. (2019). The hippocampus in depression: more than the sum of its parts? Advanced hippocampal substructure segmentation in depression. *Biol. Psychiatry* 85 487–497. 10.1016/j.biopsych.2018.08.021 30528746

[B81] Rush A. J. Fava M. Wisniewski S. R. Lavori P. W. Trivedi M. H. Sackeim H. A. (2004). Sequenced treatment alternatives to relieve depression (STAR^∗^D): rationale and design. *Control Clin. Trials* 25 119–142. 10.1016/s0197-2456(03)00112-0 15061154

[B82] Saito J. Shibasaki J. Shimokaze T. Kishigami M. Ohyama M. Hoshino R. (2016). Temporal relationship between serum levels of interleukin-6 and c-reactive protein in therapeutic hypothermia for neonatal hypoxic-ischemic encephalopathy. *Am. J. Perinatol.* 33 1401–1406. 10.1055/s-0036-1583192 27167641

[B83] Santiago G. T. P. de Menezes Galvão A. C. de Almeida R. Mota-Rolim S. A. Palhano-Fontes F. Maia-de-Oliveira J. P. (2020). Changes in Cortisol but Not in Brain-Derived Neurotrophic Factor Modulate the Association Between Sleep Disturbances and Major Depression. *Front. Behav. Neurosci.* 14:44. 10.3389/fnbeh.2020.00044 32410966 PMC7199815

[B84] Schmidt H. D. Shelton R. C. Duman R. S. (2011). Functional biomarkers of depression: diagnosis, treatment, and pathophysiology. *Neuropsychopharmacology* 36 2375–2394. 10.1038/npp.2011.151 21814182 PMC3194084

[B85] Schmitt K. Holsboer-Trachsler E. Eckert A. B. D. N. F. (2016). in sleep, insomnia, and sleep deprivation. *Ann. Med.* 48 42–51. 10.3109/07853890.2015.1131327 26758201

[B86] Schüle C. (2007). Neuroendocrinological mechanisms of actions of antidepressant drugs. *J. Neuroendocrinol.* 19 213–226. 10.1111/j.1365-2826.2006.01516.x 17280595

[B87] Sen S. Duman R. Sanacora G. (2008). Serum brain-derived neurotrophic factor, depression, and antidepressant medications: meta-analyses and implications. *Biol. Psychiatry* 64 527–532. 10.1016/j.biopsych.2008.05.005 18571629 PMC2597158

[B88] Sheline Y. I. Liston C. McEwen B. S. (2019). Parsing the hippocampus in depression: chronic stress, hippocampal volume, and major depressive disorder. *Biol. Psychiatry* 85 436–438. 10.1016/j.biopsych.2019.01.011 30777168

[B89] Shirayama Y. Yang C. Zhang J. C. Ren Q. Yao W. Hashimoto K. (2015). Alterations in brain-derived neurotrophic factor (BDNF) and its precursor proBDNF in the brain regions of a learned helplessness rat model and the antidepressant effects of a TrkB agonist and antagonist. *Eur. Neuropsychopharmacol.* 25 2449–2458. 10.1016/j.euroneuro.2015.09.002 26419294

[B90] Silverstone P. H. Entsuah R. Hackett D. (2002). Two items on the Hamilton Depression rating scale are effective predictors of remission: comparison of selective serotonin reuptake inhibitors with the combined serotonin/norepinephrine reuptake inhibitor, venlafaxine. *Int. Clin. Psychopharmacol.* 17 273–280. 10.1097/00004850-200211000-00002 12409680

[B91] Slavich G. M. Irwin M. R. (2014). From stress to inflammation and major depressive disorder: a social signal transduction theory of depression. *Psychol. Bull.* 140 774–815. 10.1037/a0035302 24417575 PMC4006295

[B92] Sousa M. B. C. Silva H. P. A. Galvão-Coelho N. L. (2015). Stress response: I. Homeostasis and allostase theory. *Estudos de Psicologia* 20 2–11. 10.5935/1678-4669.20150002

[B93] Sriram K. Rodriguez-Fernandez M. Doyle I. I. I. F. J. (2012). Modeling cortisol dynamics in the neuro-endocrine axis distinguishes normal, depression, and post-traumatic stress disorder (PTSD) in humans. *PLoS Comput. Biol.* 8:e1002379. 10.1371/journal.pcbi.1002379 22359492 PMC3280965

[B94] Steiger A. (2003). Sleep and endocrinology. *J. Intern. Med.* 254 13–22. 10.1046/j.1365-2796.2003.01175.x 12823639

[B95] Stetler C. Miller G. E. (2005). Blunted cortisol response to awakening in mild to moderate depression: regulatory influences of sleep patterns and social contacts. *J. Abnorm. Psychol.* 11 1181–1189. 10.2147/NDT.S62289 16351390

[B96] Tu M. T. Zunzunegui M. V. Guerra R. Alvarado B. Guralnik J. M. (2013). Cortisol profile and depressive symptoms in older adults residing in Brazil and in Canada. *Aging Clin. Exp. Res.* 25 527–537. 10.1007/s40520-013-0111-0 23959958

[B97] van Dalfsen J. H. Markus C. R. (2018). The influence of sleep on human hypothalamic-pituitary-adrenal (HPA) axis reactivity: a systematic review. *Sleep Med. Rev.* 39 187–194. 10.1016/j.smrv.2017.10.002 29126903

[B98] Verduijn J. Milaneschi Y. Schoevers R. A. van Hemert A. M. Beekman A. T. Penninx B. W. (2015). Pathophysiology of major depressive disorder: mechanisms involved in etiology are not associated with clinical progression. *Transl. Psychiatry* 5:e649. 10.1038/tp.2015.137 26418277 PMC5545638

[B99] Vigo D. Thornicroft G. Atun R. (2016). Estimating the true global burden of mental illness. *Lancet Psychiatry* 3 171–178. 10.1016/S2215-0366(15)00505-2 26851330

[B100] Vreeburg S. A. Hoogendijk W. J. DeRijk R. H. van Dyck R. Smit J. H. Zitman F. G. (2013). Salivary cortisol levels and the 2-year course of depressive and anxiety disorders. *Psychoneuroendocrinology* 38 1494–1502. 10.1016/j.psyneuen.2012.12.017 23313277

[B101] Vu D. Student F. Y. M. (2009). Molecular Mechanism of Depression: a narrative review of the leading neurobiological theories of Depression. *Aust. Med. Stud. J.* 9:50.

[B102] World Health Organization(Who). (2017). *Depression And Other Common Mental Disorders: Global Health Estimates.* Geneva: World Health Organization.

[B103] Yehuda R. Engel S. M. Brand S. R. Seckl J. Marcus S. M. Berkowitz G. S. (2005). Transgenerational effects of posttraumatic stress disorder in babies of mothers exposed to the World Trade Center attacks during pregnancy. *J. Clin. Endocrinol. Metab.* 90 4115–4118. 10.1210/jc.2005-0550 15870120

[B104] Yiallouris A. Tsioutis C. Agapidaki E. Zafeiri M. Agourdis A. P. Ntourakis D. (2019). Adrenal Aging and Its Implications on Stress Responsiveness in Humans. *Front. Endocrinol.* 10:54. 10.3389/fendo.2019.00054 30792695 PMC6374303

[B105] Yoshida T. Ishikawa M. Niitsu T. Nakazato M. Watanabe H. Shiraishi T. (2012). Decreased serum levels of mature brain-derived neurotrophic factor (BDNF), but not its precursor proBDNF, in patients with major depressive disorder. *PLoS One* 7:e42676. 10.1371/journal.pone.0042676 22880079 PMC3411809

[B106] Zhao G. Zhang C. Chen J. Su Y. Zhou R. Wang F. (2017). Ratio of mBDNF to proBDNF for differential diagnosis of major depressive disorder and bipolar depression. *Mol. Neurobiol.* 54 5573–5582. 10.1007/s12035-016-0098-6 27613282

[B107] Zhou C. Zhong J. Zou B. Fang L. Chen J. Deng X. (2017). Meta-analyses of comparative efficacy of antidepressant medications on peripheral BDNF concentration in patients with depression. *PLoS One* 12:e0172270. 10.1371/journal.pone.0172270 28241064 PMC5328267

[B108] Zou W. Feng R. Yang Y. (2018). Changes in the serum levels of inflammatory cytokines in antidepressant drug-naïve patients with major depression. *PLoS One* 13:e0197267. 10.1371/journal.pone.0197267 29856741 PMC5983476

[B109] Zunszain P. A. Anacker C. Cattaneo A. Carvalho L. A. Pariante C. M. (2011). Glucocorticoids, cytokines and brain abnormalities in depression. *Prog. Neuropsychopharmacol. Biol. Psychiatry* 35 722–729. 10.1016/j.pnpbp.2010.04.011 20406665 PMC3513408

