# Supplementary material for: Pathophysiology of Major Depression by Clinical Stages
Source: Front Psychol. 2021 Aug 5;12:641779. doi: 10.3389/fpsyg.2021.641779 (PMC8374436; doi:10.3389/fpsyg.2021.641779)
Supplement: Supplementary file 1 [file Table_1.pdf]

Supplementary Information (SI)

Table S1. Individual, social and clinical characteristics of volunteers.

		**MD**	**CG1**	**TRD**	**CG2**
**Sample (n)**		30	32	28	30
**Gender**	Women	16 (53.33%)	17 (53.12%)	21 (75%)	23 (76.67%)
	Men	14 (46.67%)	15 (46.88%)	7 (25%)	7 (23.33%)
**Age (years)**		μ = 24.2 ± 3.84	μ = 27.06 ± 6.42	μ = 41.57 ± 11.61	μ = 32.34 ± 1.95
**Education**	Undergraduate	18 (60%)	17 (53.13%)	22 (78.57%)	12 (40%)
	Graduate	12 (40%)	15 (46.87%)	6 (21.43%)	18 (60%)
**Familiar Income**	Low	23 (76.67%)	18 (56.25%)	23 (82.14%)	14 (46.67%)
	Medium	7 (23.33%)	14 (43.75%)	5 (17.86%)	16 (53.33%)
	High	0 (0%)	0 (0%)	0 (0%)	0 (0%)
**Clinical Aspects**	HAM-D	μ = 12.56 ± 0.56	μ = 0.71 ± 0.22	μ = 21.57 ± 0.99	μ = 0.55 ± 0.16
	Duration of disease	0	0	μ = 10.71 ± 1.83	0
	Episodes	0	0	μ = 12.71 ± 1.83	0
	Personality disorders comorbidity	0 (0%)	0 (0%)	20 (71.42%)	0 (0%)
	Anxiety comorbidity	1 (3.33%)	0 (0%)	12 (42.86%)	0 (0%)
**BMI (kg/m²)**		μ = 25.65 ± 5.42	μ = 23.98 ± 4.78	μ = 27.62 ± 1.07	μ = 23.43 ± 0.71
		U = 369 p = 0.23		U = 211 p = 0.001	
**PSQI**		μ = 9.90 ± 0.63	μ = 5.65 ± 0.52	μ = 14 ± 0.70	μ = 4.96 ± 0.43
		U = 164.5 p < 0.001		U = 8.5 p < 0.001	

MD: first episode depressive patient; CG1: control group 1; TRD: treatment-resistant depressive patients; CG2: control group 2; HAM-D: Hamilton depression scale; BMI: body mass index; PSQI: Pittsburgh Sleep Quality Index.
