# Supplementary material for: Pathophysiology of Major Depression by Clinical Stages
Source: Front Psychol. 2021 Aug 5;12:641779. doi: 10.3389/fpsyg.2021.641779 (PMC8374436; doi:10.3389/fpsyg.2021.641779)
Supplement: Supplementary file 2 [file Table_2.pdf]

Supplementary Information (SI)

Table S2. Statistical values of MANCOVA for serum cortisol (SC), salivary cortisol awakening response (CAR) and serum mature brain-derived neurotrophic factor (mBDNF) and values of GLM for C-reactive protein (CRP)

	F	p-values
**MD x CG1**		
**CAR*SC*mBDNF**		
Group	**7.56**	**< 0.001**
Age	1.51	0.19
**CAR**		
Group	**27.61**	**< 0.001**
Age	1.64	0.20
**SC**		
Group	**19.44**	**< 0.001**
Age	**5.03**	**0.02**
**mBDNF**		
Group	2.73	0.10
Age	0.66	0.41
**TRD x CG2**		
**CAR*SC*mBDNF**		
Group	**3.32**	**0.008**
Age	0.53	0.78
BMI	1.82	0.11
Income	1.78	0.78
**CAR**		
Group	**5.39**	**0.02**
Age	0.005	0.93
BMI	1.63	0.20
Income	0.10	0.74
**SC**		
Group	**6.27**	**0.01**
Age	0.72	0.39
BMI	**6.05**	**0.01**
Income	0.002	0.95
**mBDNF**		
Group	**7.58**	**0.008**
Age	0.006	0.93
BMI	2.09	0.15
Income	**4.40**	**0.04**
**CRP**		
Group	5.11	0.28
Age	0.48	0.49
BMI	**14.65**	**< 0.001**
Income	0.48	0.49

The bold text indicates statistically significant, $p < 0.05$. BMI: body mass index; MD: first episode depressive patients; TRD: treatment-resistant major depression; CG1: control group 1; CG2: control group 2.
