# Supplementary material for: Pathophysiology of Major Depression by Clinical Stages
Source: Front Psychol. 2021 Aug 5;12:641779. doi: 10.3389/fpsyg.2021.641779 (PMC8374436; doi:10.3389/fpsyg.2021.641779)
Supplement: Supplementary file 3 [file Table_3.pdf]

Supplementary Information (SI)

Table S3. Qualitative data of physiological parameters and sleep quality of depressive (MD: n = 30; TRD: n = 28) and controls groups (CG1: n = 32; CG2: n = 30).

	**CAR (cm³)**	**SC (µg/dL)**	**mBDNF (pg/mL)**	**CRP (mg/L)**
**MD**	µ = 3215.84 ± 466.93<br>µ = 3.30 ± 0.08*	µ = 431.09 ± 42.87<br>µ = 2.59 ± 0.03*	µ = 1489.84 ± 143.70	-
**CG1**	µ = 844.76 ± 165.92<br>µ = 2.79 ± 0.05*	µ = 264.80 ± 39.43<br>µ = 2.31 ± 0.05*	µ = 1833.32 ± 148.43	-
**TRD**	µ = 563.49 ± 100.67<br>µ = 2.58 ± 0.07*	µ = 151.24 ± 12.69<br>µ = 2.13 ± 0.03*	µ = 2519.40 ± 125.34	M = 3.10<br>Q25% = 0.53<br>Q75% = 5.35
**CG2**	µ = 600.06 ± 34.00<br>µ = 2.75 ± 0.02*	µ = 229.47 ± 26.75<br>µ = 2.28 ± 0.04*	µ = 2084.02 ± 105.33	M = 1.16<br>Q25% = 0.4<br>Q75% = 1.20

CAR: cortisol awakening response; SC: total serum cortisol; mBDNF: mature brain-derived neurotrophic factor; PSQI: Pittsburgh sleep quality index; CRP: C-reactive protein; MD: first episode depressive; CG1: control group 1; TRD: treatment-resistant major depression; CG2: control group 2. * mean of logarithmic.
