# Supplementary figures and images for: Pathophysiology of Major Depression by Clinical Stages
Source: Front Psychol. 2021 Aug 5;12:641779. doi: 10.3389/fpsyg.2021.641779 (PMC8374436; doi:10.3389/fpsyg.2021.641779)

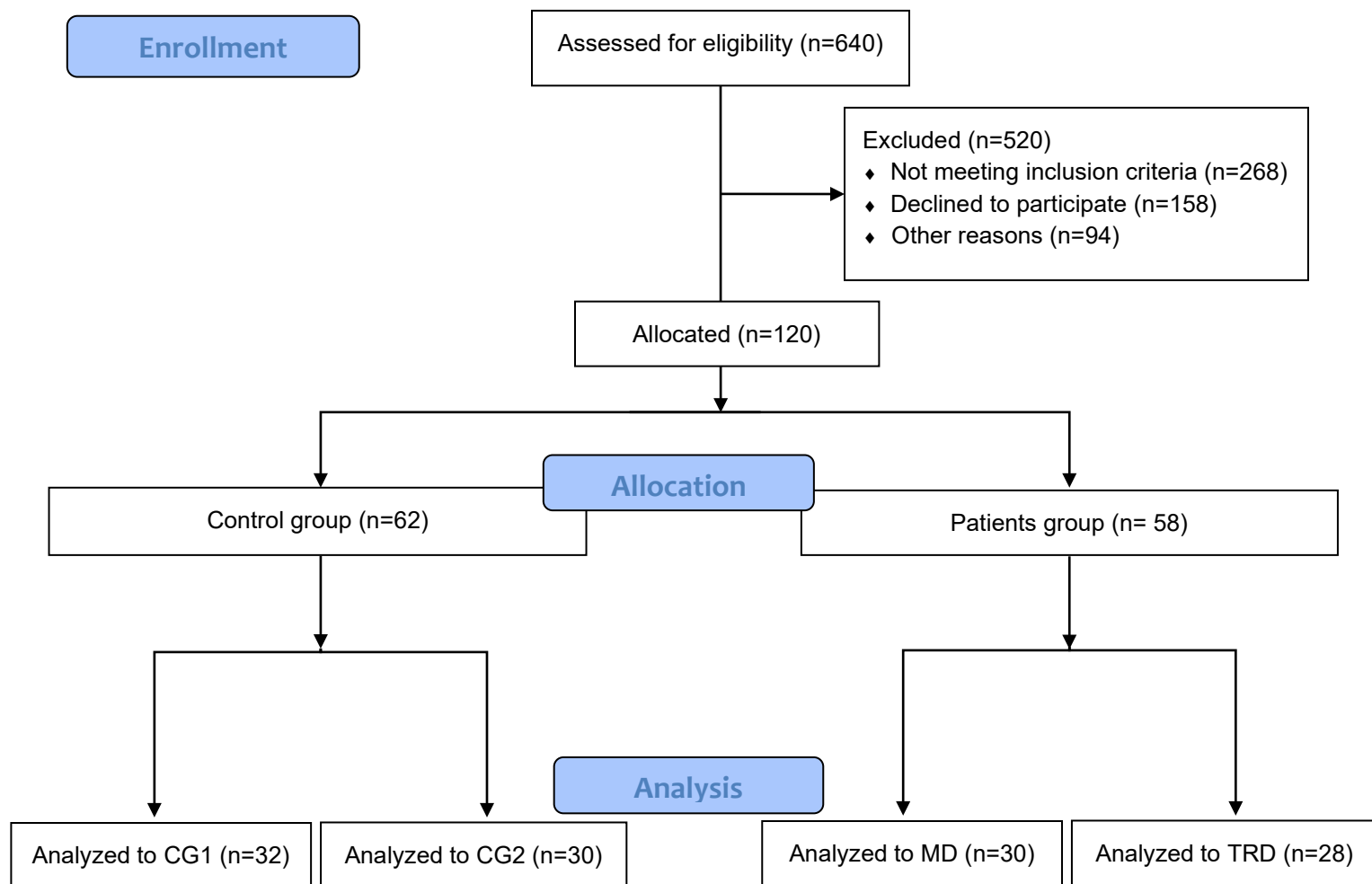

Figure S1. Flow diagram of volunteers.

Supplement: Supplementary file 4 [file Image_1.pdf]
