# Supplementary material for: Pathophysiology of Major Depression by Clinical Stages
Source: Front Psychol. 2021 Aug 5;12:641779. doi: 10.3389/fpsyg.2021.641779 (PMC8374436; doi:10.3389/fpsyg.2021.641779)
Supplement: Supplementary file 5 [file Image_2.pdf]

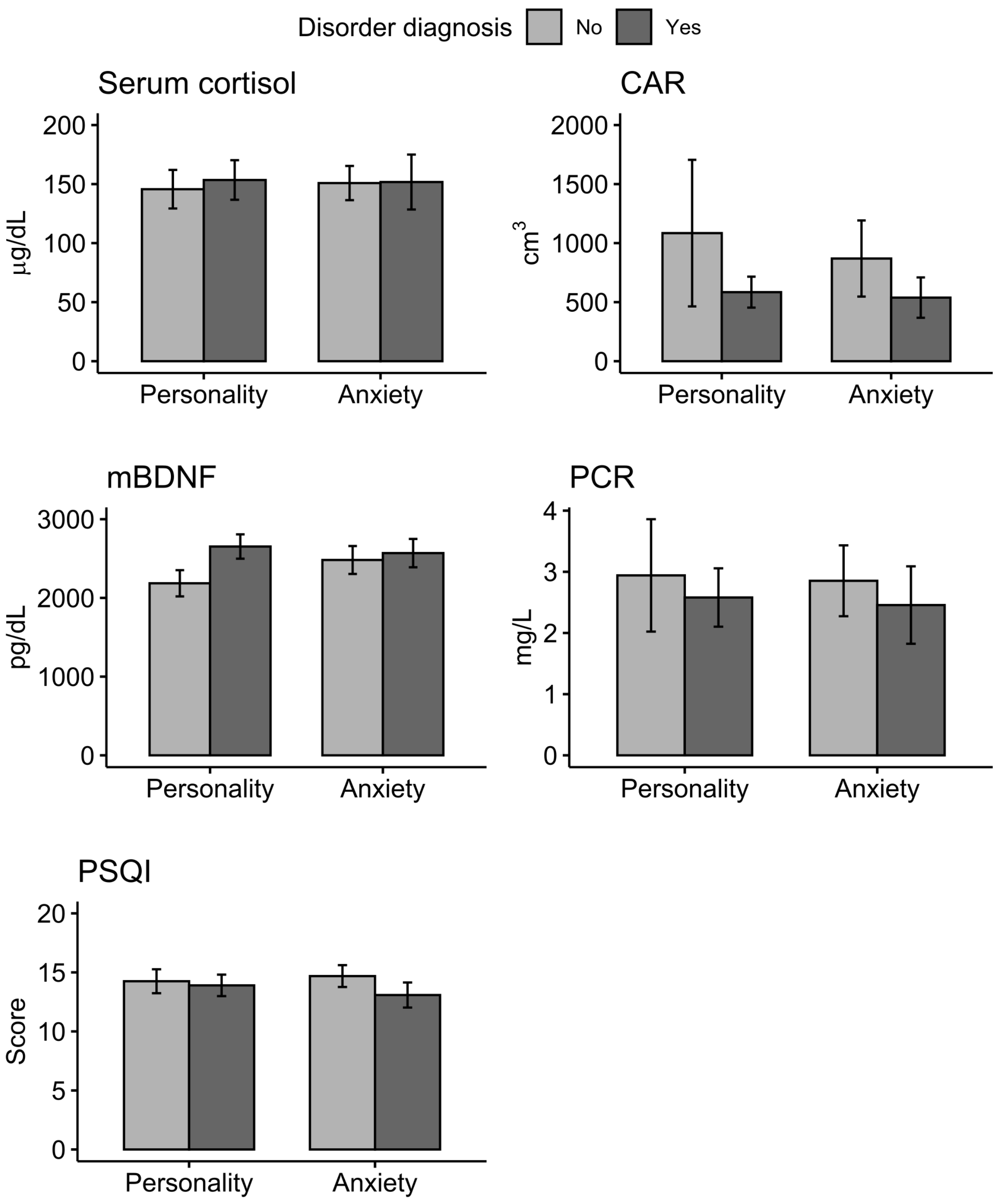

Figure S2. Clinical aspects for personality disorders and anxiety disorder for serum cortisol (SC), salivary cortisol awakening response (CAR), serum mature brain-derived neurotrophic factor (mBDNF) and Pittsburgh Sleep Quality Index (PSQI).
