# Supplementary material for: Pathophysiology of Major Depression by Clinical Stages
Source: Front Psychol. 2021 Aug 5;12:641779. doi: 10.3389/fpsyg.2021.641779 (PMC8374436; doi:10.3389/fpsyg.2021.641779)
Supplement: Supplementary file 6 [file Image_3.pdf]

Supplementary Information (SI)

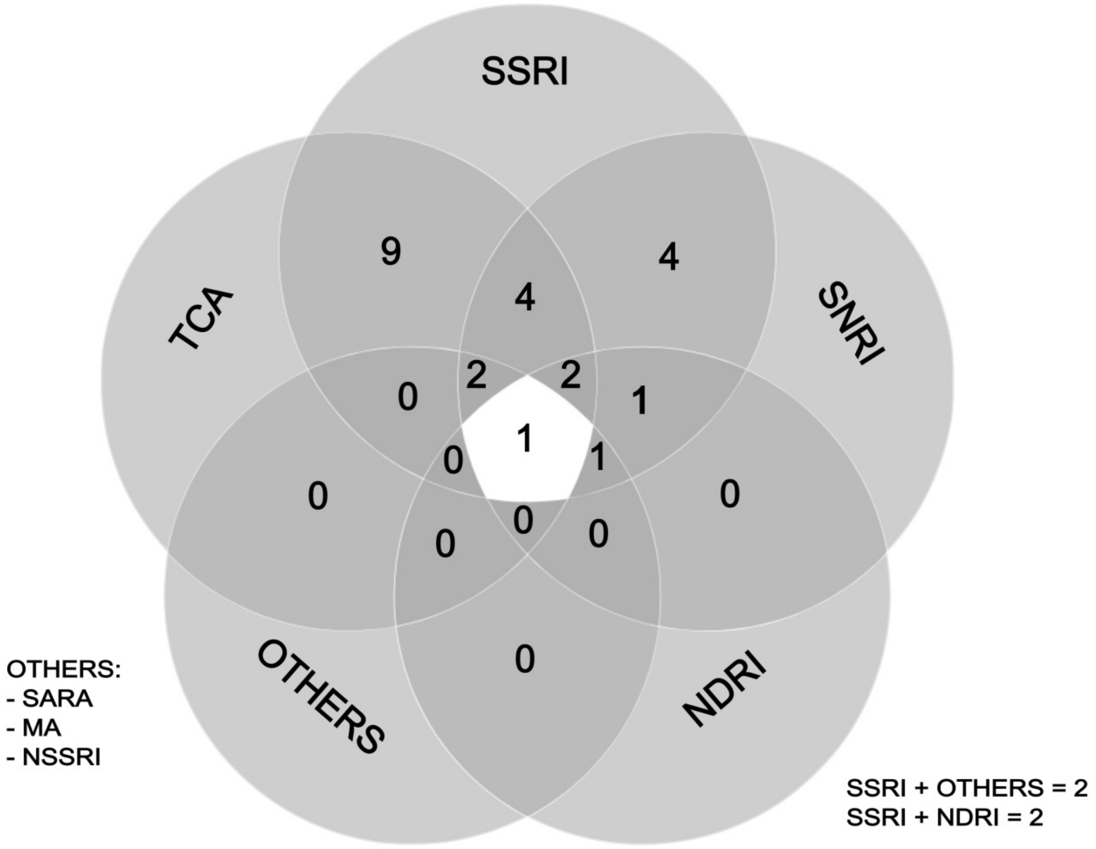

Figure S3. The classes of antidepressants used by patients with treatment-resistant major depression (TRD) before this study. 57.1% of TRD patients had used around two and three unsuccessful previous antidepressants, 28.6% of them did 4-5 unsuccessful different pharmacological treatments, and 14.3% of TRD had failed to respond to 6 -9 drugs. All patients were treated with selective serotonin reuptake inhibitors (SSRI). The tricyclic antidepressant (TCA) was the second class most used.
