# Supplementary material for: Pathophysiology of Major Depression by Clinical Stages
Source: Front Psychol. 2021 Aug 5;12:641779. doi: 10.3389/fpsyg.2021.641779 (PMC8374436; doi:10.3389/fpsyg.2021.641779)
Supplement: Supplementary file 7 [file Image_4.pdf]

Supplementary Information (SI)

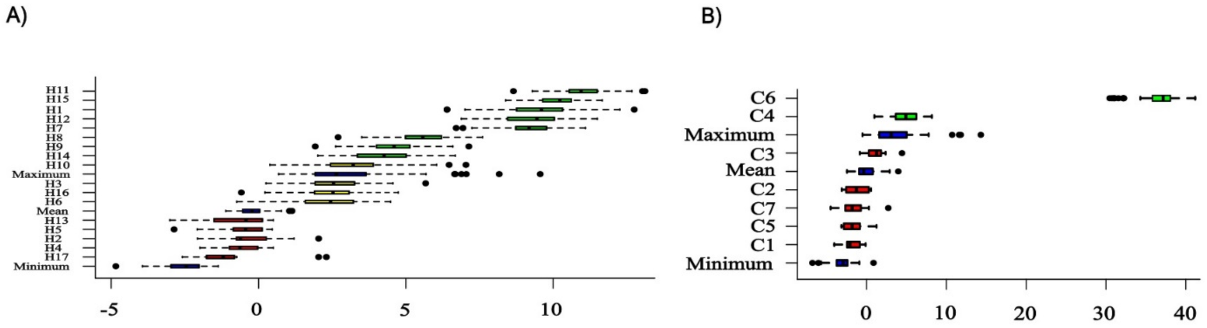

Figure S4. Random forest-based algorithm of: A) Hamilton Depression Scale (HAM-D) components of first episode depressive patients (MD: n = 30) and patients with treatment-resistant major depression (TRD: n = 28) and B) Pittsburgh Sleep Quality Index (PSQI) components of MD and TRD. Colors: green = relevant characteristic; yellow = tentative of relevance; red = no relevant characteristic; blue = randomly shuffled data at a maximum, mean and minimum level.
