# Supplementary material for: Pathophysiology of Major Depression by Clinical Stages
Source: Front Psychol. 2021 Aug 5;12:641779. doi: 10.3389/fpsyg.2021.641779 (PMC8374436; doi:10.3389/fpsyg.2021.641779)
Supplement: Supplementary file 8 [file Image_5.pdf]

Supplementary Information (SI)

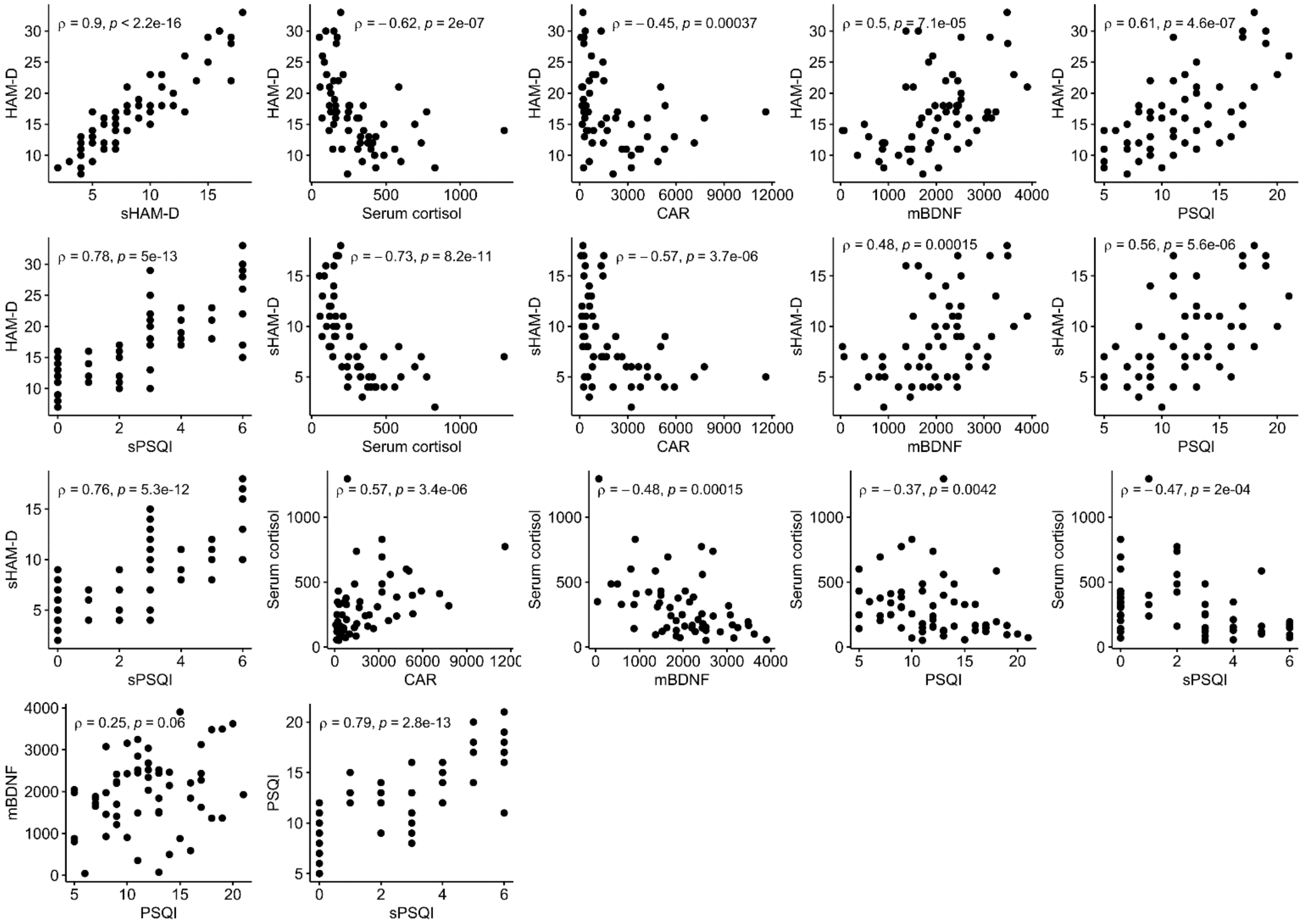

Figure S5. Scatter diagram between severity of symptoms and sleep quality with physiological parameters.
